# Effects of short-chain fatty acids on intestinal function in an enteroid model of hypoxia

**DOI:** 10.3389/fphys.2022.1056233

**Published:** 2022-12-05

**Authors:** Sarah C. Pearce, J. Philip Karl, Gregory J. Weber

**Affiliations:** ^1^ Functional Food and Nutritional Intervention Team, Combat Feeding Division, Natick, MA, United States; ^2^ Military Nutrition Division, US Army Research Institute of Environmental Medicine, Natick, MA, United States

**Keywords:** hypoxia, intestine, enteroid, short-chain fatty acid, intestinal barrier, microbial metabolites, epithelium

## Abstract

The healthy GI tract is physiologically hypoxic, but this may be perturbed by certain acute and chronic stressors that reduce oxygen availability systemically. Short-chain fatty acids have been shown to have beneficial effects on intestinal barrier function and inflammation. Therefore, our objective was to see whether short-chain fatty acids (SCFA) would improve GI barrier function, reduce production of pro-inflammatory cytokines, and increase the expression of genes regulating GI barrier function in enteroids exposed to hypoxia. Human duodenal enteroid monolayers were placed under hypoxia (1.0% O_2_) for 72 h with either 24, or 48 h pre-treatment with a high acetate ratio of SCFA’s or high butyrate ratio or placed under hypoxia concurrently. Transepithelial electrical resistance (TEER) increased with SCFA pre-treatment, especially 48 h of pre-treatment and this was maintained through the first 48 h of hypoxia while cells saw barrier function dramatically decrease by 72 h of hypoxia exposure. Inflammatory protein secretion largely decreased with exposure to hypoxia, regardless of SCFA pre-treatment. Gene expression of several genes related to barrier function were decreased with exposure to hypoxia, and with concurrent and 24 h SCFA pre-treatment. However, 48 h SCFA pre-treatment with a high butyrate ratio increased expression of several metabolic and differentiation related genes. Overall, pre-treatment or concurrent treatment with SCFA mixtures were not able to overcome the negative impacts of hypoxia on intestinal function and cells ultimately still cannot be sustained under hypoxia for 72 h. However, 48 h pre-treatment maintains TEER for up to 48 h of hypoxia while upregulating several metabolic genes.

## Introduction

The gastrointestinal (GI) tract is lined with an epithelial barrier that functions to selectively allow passage of small molecules into circulation while impeding the translocation of luminal gut microbiota and other antigens into the host circulatory system. Along this barrier, villi in the small intestine as well as crypts in both the small and large intestine maintain several epithelial cell subtypes with functions including nutrient absorption, along with hormone, anti-microbial peptide, and mucus secretion (Panwar et al., 2021). Junctional complexes positioned between adjacent epithelial cells contain tight junctions, adherens junctions, gap junctions and desmosomes (Brooke et al., 2012). These complexes along with the tight junction proteins regulate passage of molecules through paracellular pathways (France and Turner, 2017) and complement the functions of transporters and other mechanisms regulating transcellular transport of compounds from the intestinal lumen.

A healthy GI tract exists under a state of “physiologic hypoxia” characterized by a steep oxygen gradient from the submucosa to the lumen, with luminal oxygen concentrations far below the atmospheric oxygen concentration of ∼21% O_2_. (Zheng et al., 2015). This physiologic hypoxia activates transcription factors, known as hypoxia-inducible factors, which regulate pathways involved in maintaining GI epithelial barrier function by modulating tight junction function, inflammatory tone, secretion of mucin and anti-microbial compounds, and activity of epithelial nutrient transporters (Pral et al., 2021). Physiologic hypoxia at the level of the GI tract may be perturbed by acute and chronic stressors that reduce oxygen availability systemically such as high terrestrial altitude (Karl et al., 2018), intense exercise (Hill et al., 2020), and heat stress (Pearce et al., 2013) among others. Inflammation, oxidative stress, metabolic dysfunction, apoptosis and GI barrier damage can result, thereby, initiating a cycle in which translocation of antigens from the gut lumen induce immune and inflammatory responses that exacerbate GI barrier damage. This cycle is thought to contribute to decrements in physical and cognitive performance during acute hypoxic exposures (Ando et al., 2020), and development of disease under conditions of chronic intermittent hypoxia (Behrendt et al., 2022). As such, there is interest in identifying strategies that promote GI barrier resilience to the damaging effects of hypoxia.

Increasing short-chain fatty acid (SCFA) production by the gut microbiota may be one feasible strategy. SCFA are derived from fermentation of undigested carbohydrate by the gut microbiota. Acetate, propionate, and butyrate are the predominant SCFA within the lumen and are commonly found at relative proportions approximating 60:20:20 (acetate/propionate/butyrate) in concentrations that range from 10–40 mM in the small intestine to 50–150 mM in the colon (Schmitt et al., 1977; Cummings et al., 1987). Butyrate, and acetate to a lesser extent, provide an energy source for enterocytes. This promotes epithelial O_2_ consumption thereby stabilizing HIF-1α and promoting GI barrier integrity (Pral et al., 2021). Through this and other pathways, SCFA’s regulate intestinal immune function, cellular differentiation, barrier integrity, apoptosis, hormone secretion and cell metabolism (Pearce et al., 2020; Pral et al., 2021).

Favorable effects of SCFA on GI barrier function likely vary according to both the size and composition of the SCFA pool. In support, moderate SCFA concentrations of 40–80 mM ratio have shown positive effects on intestinal barrier function as measured by transepithelial electrical resistance (TEER) *in vitro*, relative to lower (20 mM) or higher (100–200 mM) concentrations (Chen et al., 2017). In addition, when multiple ratios of acetate, propionate, and butyrate at 40 mM were examined, the largest increase in TEER was found with high butyrate concentrations reaching 50% of the SCFA pool (Chen et al., 2017). That observation may be attributable to butyrate being the preferred energetic substrate for enterocytes. Indeed, butyrate has been shown to enhance epithelial barrier function in several models (Chen et al., 2017; Pearce et al., 2020; Huang et al., 2022) as well as favorably modulate inflammatory pathways within the intestinal epithelium (Yang et al., 2020).

Although SCFA favorably modulate GI barrier function under normal physiologic conditions, whether the same is true when physiologic hypoxia is disrupted by environmental and physiologic stressors that reduce systemic oxygen availability is unclear. Therefore, the objective of this study was to examine the effects of SCFA on changes in GI barrier function, cytokine production and gene expression during hypoxic stress. Two different compositions of SCFA, one reflecting the normal *in vivo* proportions with a higher ratio of acetate, propionate, and butyrate and the other reflecting a high butyrate ratio were studied. As studying human GI barrier function *in vivo* is limited by the inaccessibility of the human GI tract, we utilized an intestinal enteroid model that provides a species-specific and physiologically relevant approach to studying mechanisms influencing GI barrier function under controlled environmental conditions and interventions (Taelman et al., 2022). While enteroid models have increasingly been utilized to study the effects of SCFA’s and *in vitro* fermentation-derived microbial metabolites on the intestinal barrier (Schilderink et al., 2016; Pearce et al., 2020; Pace et al., 2021; Pearce et al., 2022), to our knowledge, these models have not been used to determine the effects of SCFA on intestinal barrier responses to hypoxic stress. We hypothesized that SCFA would improve GI barrier function, reduce the production of pro-inflammatory cytokines, and increase the expression of genes regulating GI barrier function in enteroids exposed to hypoxia and that increasing the proportion of butyrate in the SCFA pool would enhance these benefits.

## Methods

### Enteroids

De-identified endoscopic tissue biopsies were collected from grossly unaffected (macroscopically normal) areas of the duodenum in 10–14 year old patients undergoing endoscopy for gastrointestinal complaints. Informed consent and developmentally appropriate assent were obtained at Boston Children’s Hospital from the donors’ guardian and the donor, respectively. All methods were carried out in accordance with the Institutional Review Board of Boston Children’s Hospital (Protocol number IRB-P00000529) approval. Tissue was digested in 2 mg ml^−1^ collagenase I for 40 min at 37°C followed by mechanical dissociation, and isolated crypts were resuspended in growth factor-reduced Matrigel and obtained as frozen enteroids at low passage number (Kasendra et al., 2018; Zeve et al., 2022).

### 3D cultures

Frozen enteroids were thawed and immediately transferred to a 15 ml conical tube with 5 ml of complete media without growth factors (CMGF-) containing Advanced DMEM/F12, 0.2 mM Glutamax, and 10 mM HEPES. Cells were then gently spun down at 300 × *g* for 10 min and supernatant removed. Enteroid pellets were resuspended in growth-factor reduced Matrigel (#356231, Corning, Corning, NY). Aliquots (40 μl) containing ∼100 enteroids were plated in individual wells of a 24-well tissue culture treated plate and incubated at 37°C for 10 min before adding 0.5 ml of media (Human Intesticult^™^ Stem Cell Technologies, Cambridge, MA). Media was replaced every 2 days, and enteroids were passaged every 5–7 days by incubation in Gentle Cell Dissociation Reagent (Stem Cell Technologies, Cambridge, MA) at 4°C with shaking for 40 min. Well contents were scraped and triturated with a P200 pipette tip 30–50 times to break apart enteroids, collected in a 15 ml conical tube with 1:1 addition of media and centrifuged at 300 × *g* for 10 min. Cell pellets were resuspended in Matrigel to achieve a similar density each time. Experiments were conducted on enteroids between passages 10–15.

### 2D monolayer cultures

Monolayer protocols were adapted from previous publications [13]. Enteroids were initially cultured in matrigel for two to three passages prior to plating on Transwell inserts^™^. To form monolayers, Transwell^™^ inserts (24-well inserts, 0.33 cm^2^ surface area, 0.4 μm pore polyester membrane; Corning, Corning, NY) were coated with human collagen IV solution (final concentration of 10 μg/cm^2^) and incubated overnight at 4°C. Human collagen IV (Millipore Sigma, Burlington, MA) was purchased as a liquid in 0.5 M acetic acid, then diluted using sterile water. Prior to plating, any remaining collagen was removed from wells and washed ×2 with Advanced DMEM/F12. Fragments for monolayer plating were obtained using the passaging protocol above. Approximately 50 enteroid fragments were obtained per 100 μl Intesticult^™^ media, then added to the filter and allowed to settle at 37°C. 600 μl Intesticult^™^ media was also added to the basolateral side. Media was changed every 2 days and monolayer development was tracked using transepithelial electrical resistance (TEER) measured by the EVOM2 epithelial voltohmmeter with STX2 “chopstick” electrodes with a ± µA nominal at 12.5 Hz (World Precision Instruments, Sarasota, FL).

### Short-chain fatty acid treatment

Short-chain fatty acids acetate, propionate and butyrate (Sigma Aldrich, St. Louis, MO) in salt form diluted in purified water were used to treat enteroids at physiologically relevant small intestinal concentrations (40 mM) for human at physiologic proportions (aceate:propionate:butryrate; 60:20:20 ratio; ACET), or high butyrate (37.5:12.5:50 ratio; BUT). High BUT ratio was chosen based off previous research showing optimal concentrations and ratios for epithelial barrier function *in vitro* (Chen et al., 2017)*.* Enteroids were either treated with ACET or BUT while concurrently undergoing 72 h of hypoxia (Experiment 1) or were pre-treated for 24 h (Experiment 2) or 48 h (Experiment 3) prior to being put under HYP for 72 h. SCFAs were removed after either 24 or 48 h prior to undergoing hypoxia in both pre-treatment groups (Experiments 2 and 3). Three to six technical replicates were generated for each experimental condition.

### Hypoxia treatment

Cells were treated using a hypoxia *in vitro* glove box (Coy Laboratories, Grass Lake, MI) at 1% oxygen for 72 h. The oxygen concentration 1% for hypoxia was chosen based on previous standardized *in vitro* hypoxia models as well as information on physiologic hypoxia (Zheng et al., 2015; Pavlacky and Polak, 2020). Controls were kept at atmospheric oxygen concentrations (∼21%).

### RNA extraction and gene analysis

Total RNA was extracted from intestinal enteroids using a commercially available kit (RNeasy Micro, Qiagen). Purified RNA was then run on a QuantiGene Plex Gene Expression Array (ThermoFisher Scientific, Waltham, MA) and analyzed on a Luminex MAGPIX Instrument (Luminex, Northbrook, IL). Targets were designed by ThermoFisher and genes analyzed include Rab17, member RAS oncogene family (*RAB17*), Claudin-4 (*CLDN4*), Chromogranin A (*CHGA*), Doublecortin-like kinase 1 (*DCLK1*), Interleukin-22 (*IL-22*), Alkaline phosphatase, intestinal (*ALPI*), Peptide YY (*PYY*), Interleukin-8 (*IL8*), Claudin-3 (*CLDN3*), Monocarboxylate transporter 1(*SLC16A1*), Monocarboxylate transporter 4 (*SLC16A3*), Trefoil factor 3 (*TFF3*), Transforming growth factor beta-1 (*TGFB1*), Cadherin-1 (*CDH1*), Lysozyme (*LYZ*), Fas cell surface death receptor (*FAS*), Heat shock protein family A member 1A (*HSPA1A*), Heat shock factor 1 (*HSF1*), Acyl-CoA dehydrogenase medium chain (*ACADM*), Occludin (*OCLN*), Sodium-coupled monocarboxylate transporter 1 (*SLC5A8*), Tight junction protein 1 (*TJP1*), Free fatty acid receptor 2 (*FFAR2*), Myosin VIIB (*MYO7B*), Fatty acid binding protein 2 (*FABP2*),

### Secreted protein analyses

Basolateral media taken after of hypoxic exposure from organoids cultured in 2D was analyzed for secreted proteins using a custom ProcartaPlex Immunoassay Kit with antibody-based magnetic beads (ThermoFisher Scientific, Waltham MA) for the following targets: Ghrelin, Glucagon-like peptide 1 (GLP-1), Peptide YY (PYY), Interleukin 1β (IL-1β), Interleukin-4 (IL-4), Interleukin-6 (IL-6), Interleukin-8 (IL-8), Interleukin-10 (IL-10), Interleukin-12p70 (IL-12p70), Interleukin 17 (IL-17), Interleukin-18 (IL-18), Interferon gamma (IFN-γ), Monocyte chemoattractant protein-1 (MCP-1), FAS-ligand (FAS-L), Galectin-3, and Lactate dehydrogenase B (LDH-B). Samples were read and fluorescent intensity were analyzed on a Luminex MAGPIX multiplexing system (Luminex, Northbrook, IL).

### Statistical analysis

For each outcome, separate analyses were conducted for Experiments 1, 2 and 3. TEER values were log_10_-transformed and analyzed using general linear models with correlated errors that included experimental condition, time and their interaction included as fixed factors. Following significant main effects or interactions, within and between condition comparisons were conducted using paired and independent samples t-tests, respectively, and *p*-values were adjusted using a Bonferroni correction. Protein concentrations were analyzed as a standardized difference from the mean of the control condition [(treatment concentration—control mean)/control SD], and gene expression data as relative expression to the mean of the control condition [gene expression during treatment/mean gene expression during control]. Both standardized protein concentrations and relative gene expression were analyzed using one-way ANOVA with Bonferroni corrections. Data analyses were completed in SPSS v. 26.0 and *p* < 0.05 was considered statistically significant.

## Results

### Barrier function

**FIGURE 1:**
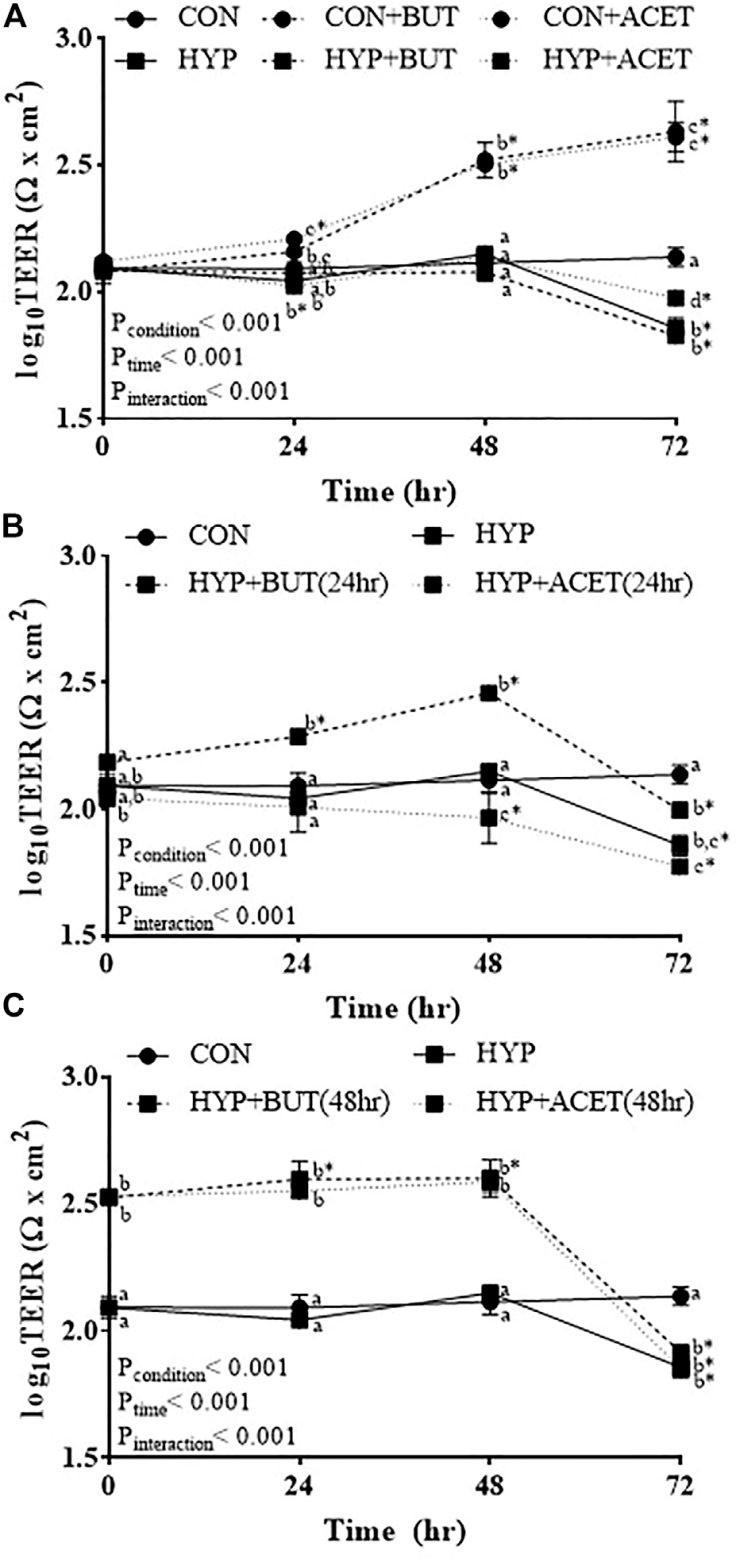
Effects of butyrate (BUT) and acetate (ACET) on transepithelial electrical resistance (TEER) during 72-hr hypoxia (HYP) exposure. **(A)** Cells treated with butyrate or acetate throughout the 72 h incubation. **(B)** Cells pre-treated with butyrate or acetate for 24 h. **(C)** Cells pre-treated with butyrate or acetate for 48 h. **(B,C)** Butyrate and acetate removed at 0 h. **(A–C)** General linear model with correlated errors and Bonferroni corrections (*n* = 3–4 per condition). Within a time point, conditions not sharing a superscript letter are significantly different (*p* < 0.05). *Within-condition difference from 0 h (*p* < 0.05). CON, control condition.

For all experiments, significant condition-by-time interactions were seen for changes in TEER (*p* < 0.001, Figures 1A–C). When cells were not treated with SCFA, TEER remained constant throughout the 72 h experimental period in the control condition (CON) but was decreased after 72 h of hypoxia exposure (HYP; Figures 1A–C).

In Experiment 1 (SCFA treatment concurrent to hypoxia exposure), SCFA treatment increased TEER relative to CON (CON + ACET and CON + BUT; Figure 1A). Concurrent treatment with ACET (HYP + ACET), but not BUT (HYP + BUT), attenuated the decrease in TEER seen in untreated cells following 72 h exposure to hypoxia (Figure 1A).

In Experiment 2 (24 h pre-treatment with SCFA), 24 h pre-treatment with BUT increased TEER during the initial 48 h of hypoxia exposure, but not after 72 h of exposure [HYP + BUT (24 h)]; Figure 1B. In contrast, 24 h pre-treatment with ACET [HYP + ACET (24 h)] resulted in a reduction in TEER after 48 h of exposure to hypoxia which was not different from HYP after 72 h of exposure (Figure 1B).

In Experiment 3 (48 h pre-treatment with SCFA), 48 h pre-treatment with both BUT [HYP + BUT (48 h)] and ACET [(ACET + BUT (48 h)] resulted in higher TEER at 0 h and after 24 and 48 h of exposure to hypoxia relative to both CON and HYP (Figure 1C). However, neither treatment attenuated the hypoxia-induced reduction in TEER observed after 72 h exposure.

### Protein secretion

**FIGURE 2:**
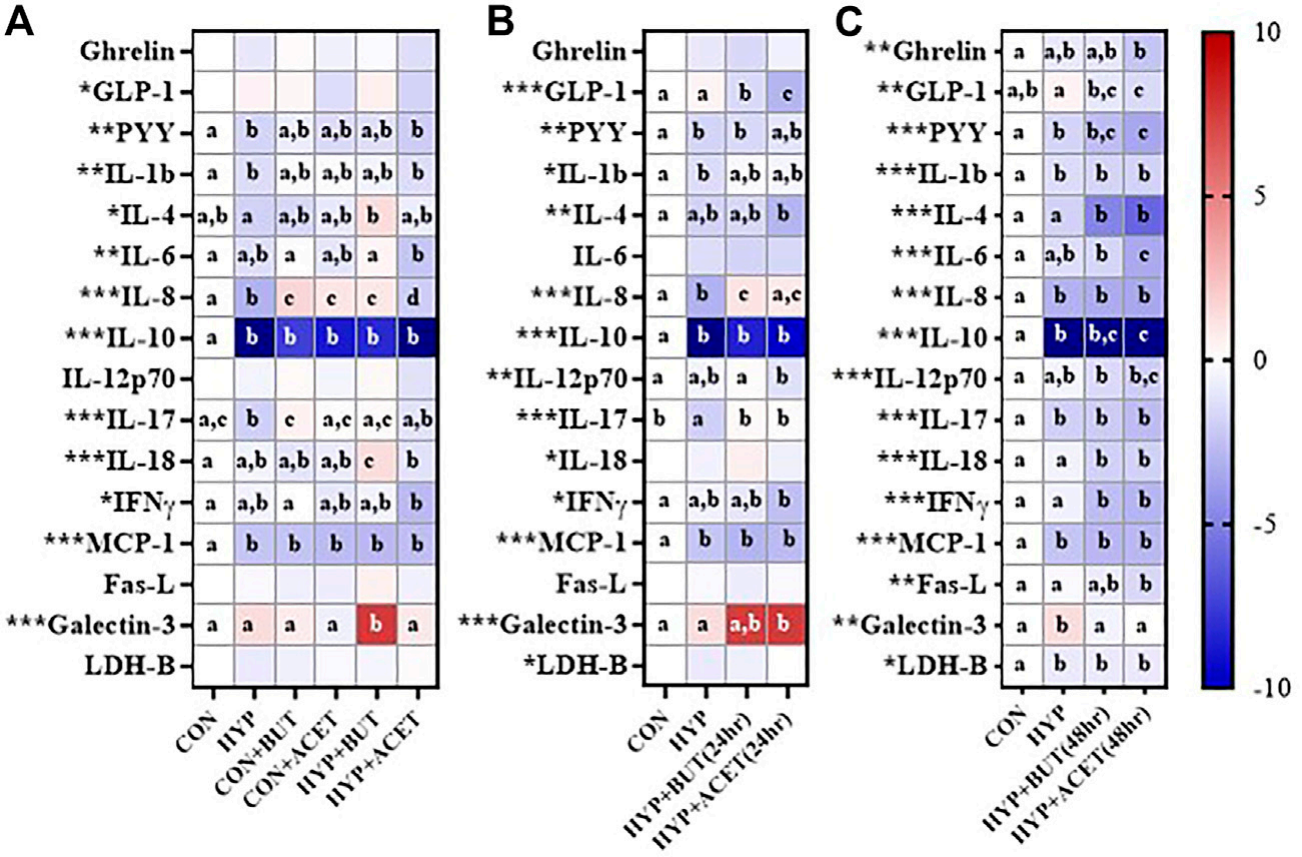
Effects of butyrate (BUT) and acetate (ACET) on protein concentrations during 72-h hypoxia (HYP) exposure. **(A)** Cells treated with butyrate or acetate thrhoughout the 72 h incubation. **(B)** Cells pre-treated with butyrate or acetate for 24 h. **(C)** Cells pre-treated with butyrate or acetate for 48 h. **(B,C)** Butyrate and acetate removed at 0 h. **(A–C)** Heatmap represents mean standardized difference from control condition (CON). One-way ANOVA with Bonferroni corrections (*n* = 6 per condition). *p < 0.05; **p < 0.01; ***p < 0.001. For each outcome, conditions not sharing a superscript letter are significantly different (*p* < 0.05).

Hypoxia without SCFA treatment reduced concentrations of several proteins relative to CON including PYY, IL-1β, IL-8, IL-10, IL-17, and MCP-1 (Figure 2A).

In Experiment 1, concurrent ACET (CON + ACET) and BUT (CON + BUT) treatment without hypoxia increased IL-8 concentrations and decreased IL-10 and MCP-1 concentrations relative to CON (Figure 2A). The effect of ACET and BUT treatment on IL-8 was also seen during hypoxia treatment (HYP + ACET, HYP + BUT; Figure 2A). BUT treatment concurrent to hypoxia (HYP + BUT) also increased IL-18 and galectin-3 compared to all other groups (Figure 2A).

In Experiment 2, 24 h pre-treatment with both ACET [HYP + ACET (24 h)] and BUT [HYP + BUT (24 h)] prevented hypoxia-induced decreases in IL-8 and IL-17 concentrations and decreased GLP-1 concentrations relative to both HYP and CON (Figure 2B). IFNγ, IL-12p70, and IL-4 were all significantly decreased, and galectin-3 was increased, in HYP + ACET (24 h) but not HYP + BUT (24 h) compared to CON (Figure 2B).

In Experiment 3, 48 h of pre-treatment with both ACET [HYP + ACET (48 h)] and BUT [HYP + BUT (48 h)] significantly decreased concentrations of several proteins compared to both CON and HYP groups including IL-4, IL-18, and IFN-γ (Figure 2C). In addition, IL-1β, IL-8, IL-10, IL-17, MCP-1, and LDH-B were all significantly decreased in both groups compared to CON but were not different from HYP (Figure 2C).

Measured protein concentrations are provided in Supplementary Table S1.

### Gene expression

**FIGURE 3:**
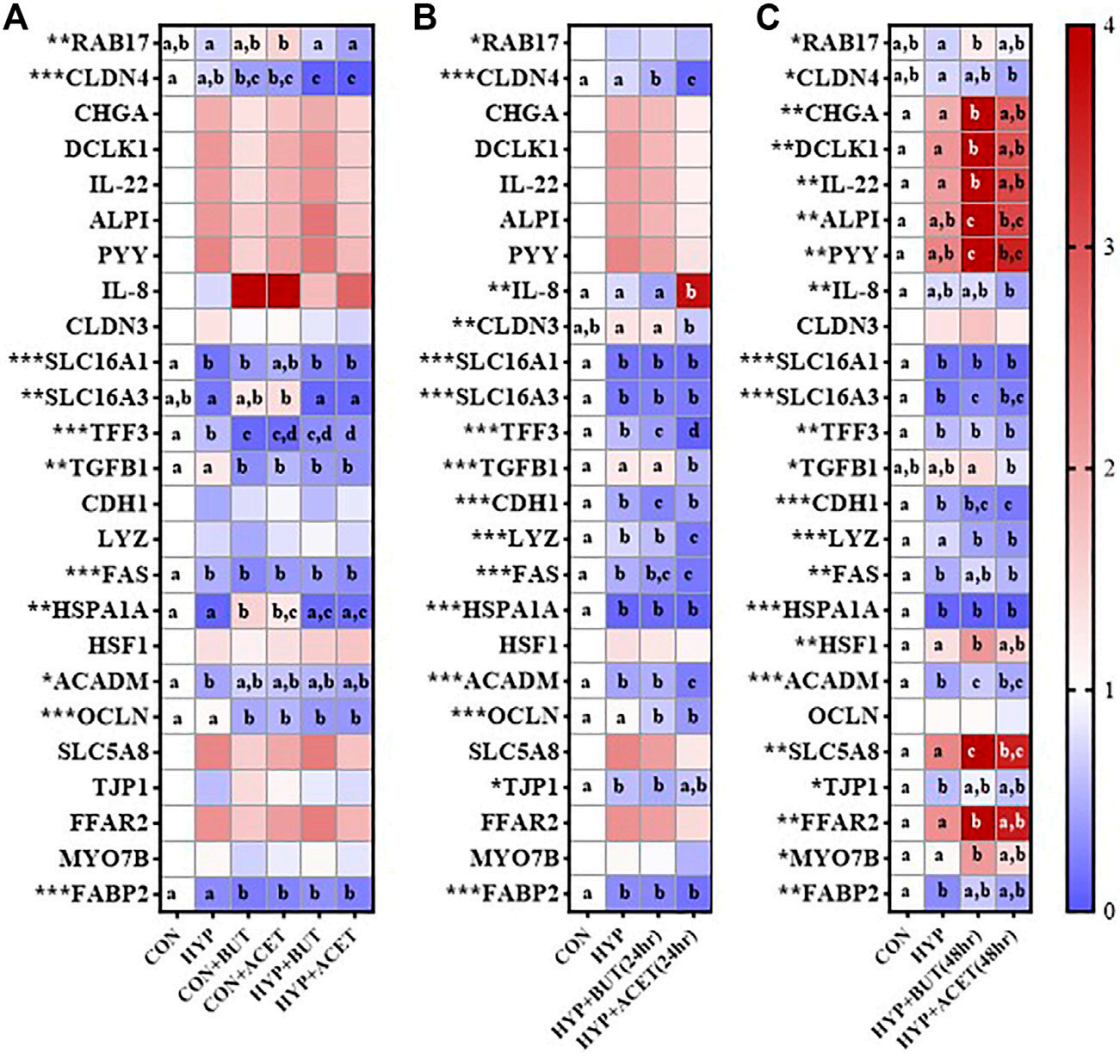
Effects of butyrate (BUT) and acetate (ACET) on gene expression during 72-h hypoxia (HYP) exposure. **(A)** Cells treated with butyrate or acetate throughout the 72 h incubation. **(B)** Cells pre-treated with butyrate or acetate for 24 h. **(C)** Cells pre-treated with butyrate or acetate for 48 h. **(B,C)** Butyrate and acetate removed at 0 h. **(A–C)** Heatmap represents mean expression relative to control condition (CON). One-way ANOVA with Bonferroni corrections (*n* = 3–4 per condition). *p < 0.05; **p < 0.01; ***p < 0.001. For each outcome, conditions not sharing a superscript letter are significantly different (*p* < 0.05).

Hypoxia without SCFA treatment reduced expression of *FAS*, *ACADM*, *FABP2*, *SLC16A1,* and *TFF3* compared to CON (Figure 3A).

In Experiment 1, concurrent treatment of CON with both ACET (CON + ACET) and BUT (CON + BUT), decreased expression of *CLDN4*, *TFF3*, *TGFB1*, *FAS*, *OCLN*, and *FABP2* and increased expression of *HSPA1A* relative to CON (Figure 3A). These effects were largely unaffected by hypoxia exposure (HYP + ACET and HYP + BUT). However, hypoxia did influence the effects of ACET on expression of *RAB17* and *SLC16A3* and the effects of BUT on *HSPA1A* (Figure 3A).

In Experiment 2, 24 h pre-treatment with ACET [HYP + ACET (24 h)] reduced expression of *TFF2*, *TGFB1*, *LYZ*, *FAS*, *ACADM*, *OCLN*, *CLDN4* and *CLDN3*, and increased expression of IL-8 relative to hypoxia alone (Figure 3B). 24 h pre-treatment with BUT [HYP + BUT (24 h)] reduced expression of *CLDN4*, *TFF*, *CDH1* and *OCLN* relative to hypoxia alone (Figure 3B).

In Experiment 3, 48 h pre-treatment with BUT for 48 h [HYP + BUT (48 h)] increased *CHGA*, *DCLK1*, *IL-22*, *ALPI*, *PYY*, *SLC5A8*, *HSF1*, *FFAR2*, and *MYO7B* relative expression compared to CON and HYP (Figure 3C). The same effects were not seen for 48 h pre-treatment with ACET [HYP + ACET (48 h)] with the exception of *SLC5A8* (Figure 3C).

Tables with full relative expression values are provided in Supplementary Table S2.

## Discussion

SCFA’s favorably modulate GI barrier function under normal physiologic conditions, but whether the same is true when challenged by environmental and physiologic stressors that reduce systemic oxygen availability is unclear. Therefore, this study examined whether SCFA’s could provide protection against environmental hypoxia-induced epithelial damage in an *in vitro* model. One caveat of this study is that duodenal enteroids were used. Though these cells are more sensitive to oxygen changes and have significantly less exposure to SCFA than colonocytes, enterocytes within the ileum are also exposed to microbiota derived SCFA (Parada Venegas et al., 2019a). An additional caveat is that we used standard oxygen concentrations for controls and hypoxia treatments. We recognize that as research continues to advance, studies are moving towards more physiologically relevant conditions (Skovdahl et al., 2021) and models (Kasendra et al., 2018). Herein, we demonstrate that hypoxia exposure decreased enteroid epithelial barrier integrity, after 48 h. Notably, treatment with a high butyrate but not high acetate SCFA mixture concurrent to hypoxic-stress and for 24 h prior to hypoxic-stress attenuated, but did not prevent, hypoxia-induced epithelial barrier dysfunction. Additionally, pre-treatment with SCFA mixtures proportionally high in either butyrate or acetate for 48 h prior to hypoxic-stress transiently enhanced epithelial barrier integrity during hypoxic stress before the benefit was subsequently lost. Collectively observations: 1) confirm that SCFA enhance epithelial barrier integrity *in vitro*, 2) demonstrate that hypoxia can damage epithelial barrier integrity *in vitro*, 3) suggest that increasing SCFA production prior to hypoxic stress could transiently strengthen epithelial barrier integrity during hypoxic-stress, and 4) indicate that SCFA-derived benefits on epithelial barrier function during hypoxia might be improved by increasing the proportion of butyrate within the SCFA pool. Whether maintaining SCFA treatments prior to and throughout hypoxic exposures can extend protection epithelial barrier protection beyond the initial 48 h of hypoxic stress should be addressed in future research.

SCFA concentrations (Parada Venegas et al., 2019b) are lower in the small intestine than the large intestine, and the small intestine may be more sensitive to changes in oxygen due to higher basal oxygen concentrations (Konjar et al., 2021). The SCFA butyrate is a primary energy source of intestinal epithelial cells and is utilized *via* β-oxidation which accounts for ∼75% of oxygen consumption in colonocytes (Roediger, 1980). This process also helps maintain an anaerobic oxygen gradient of <1% in the lumen. After transport into the cells, butyrate enhances oxidative phosphorylation, which consumes oxygen (Kelly et al., 2015). Butyrate has been shown to enhance epithelial barrier function in several models (Chen et al., 2017; Pearce et al., 2020; Huang et al., 2022) as well as modulate inflammatory pathways (Yang et al., 2020).

Results confirmed that SCFA enhance intestinal barrier function *in vitro* under normoxic conditions, and this is similar to previous research in our laboratory using single SCFA (Pearce et al., 2020). Further, pre-treatment of enteroids with SCFA for 48 h increased TEER dramatically and enabled cells to maintain a high TEER until 72 h of hypoxia treatment, potentially providing some level of protection during hypoxic exposure. However, SCFA, whether provided at physiologic ratios or an enhanced butyrate ratio, and whether provided concurrent to or as a pre-treatment, did not prevent hypoxia-mediated decreases in GI barrier function after 72 h of exposure. It is, however, worth noting that barrier function was maintained up through 48 h of hypoxic exposure, which was especially apparent in the 48 h pre-treatment groups. In contrast, concurrent treatment with SCFA’s during hypoxia does not appear to be long enough to prime an epithelial barrier response as TEER remains stable and then eventually decreases, as opposed to increasing initially.

Protein concentrations and gene expression were measured to identify mechanisms through which SCFA may affect epithelial barrier function during hypoxic stress. Unfortunately, these measurements were taken after 72 h exposure to hypoxia, a time point at which TEER demonstrated few, if any, differences amongst the hypoxia conditions. It is therefore unsurprising that major differences in protein and gene expression were not observed.

One exception was IL-8. Notably, using the same enteroid model, our group has also previously demonstrated an increase in IL-8 due to single SCFA exposure (Pearce et al., 2020) and due to a milleu of fecal metabolites including SCFA (Pearce et al., 2022). In the current study, a significant increase in IL-8 was also observed at the protein level, in addition to a large numeric difference at the gene level with both high butyrate and high acetate treatments. Increased IL-8 has been associated with decreased TEER in previous studies (Ko et al., 2007) as it is an epithelial-produced chemokine which recruits immune cells to the subepithelial area and plays a role in bacterial translocation across the epithelium (Sansonetti et al., 1999). In the current study, we show that IL-8 protein is increased only in treatment groups with the high butyrate ratio under concurrent and 24-h pretreatment. However, with 48 h pre-treatment this trend reverses and IL-8 is increased due to SCFA-treatment alone, regardless of hypoxia. Thus, the current mechanism is unclear. That gene expression patterns differed from protein concentrations may be an effect of timing of sample collection.

Hypoxia and SCFA mixtures also strongly inhibited secretion of the anti-inflammatory cytokine IL-10. This may provide insight into the lack of protection against barrier dysfunction in the hypoxic models. IL-10 plays an important role as a positive regulator of the NFκB pathway and intestinal homeostasis (Papoutsopoulou et al., 2021). In addition, loss of the IL-10 receptor disrupts intestinal cell fate and differentiation (Jenkins et al., 2021).

Hypoxia alone appeared to generally reduce cytokine secretion. SCFA did not appear to affect that reduction except for IL-8 and IL-17, which tended to increase with SCFA treatment and hypoxia in the concurrent and 24 h treatments. SCFA are known to act on immune cells including production of multiple cytokines including TNF-α, IL-6, and IL-10 as well as certain chemokines (Vinolo et al., 2011). In this study BUT appears to be initiating a more pro-inflammatory phenotype without decreasing TEER which could be due to a compensatory response. Research in endothelial cells has shown that hypoxia induces TNF-α which can then in turn upregulate the HIF pathway which may also be a survival mechanism (Jin et al., 2019).

Gene expression results show general downregulated expression patterns for most genes under hypoxic conditions alone, as well as under concurrent treatment and 24 h pre-treatment. Interestingly, several genes were upregulated with 48 h SCFA pre-treatment, especially in the higher butyrate ratio treatment. Upregulated genes were largely related to metabolism. For example, a transporter (SLC5A8) which is known to transport butyrate (Dang et al., 2021) was upregulated in the 48 h BUT pre-treatment groups. SCFA responsive free fatty acid receptor FFAR2 (Pan et al., 2018) was also increased in the same group along with enteroendocrine cell markers CHGA and PYY, and enterocyte enzyme alkaline phosphatase which have all been previously shown to be responsive to butyrate (Pearce et al., 2020). These are likely upregulated due to the 48 h pre-treatment with the high butyrate ratio. It is possible that because only one time point at 72 h was measured that the upregulation of additional genes at earlier time points may have been missed.

Collectively, these observations suggest that a high butyrate ratio may be more effective than a high acetate ratio for attenuating hypoxia induced intestinal barrier dysfunction, but that any benefits are short-lived. Overall, pre-treatment or concurrent treatment with SCFA mixtures were not able to overcome the negative impacts of hypoxia on intestinal function and cells ultimately could not be sustained under hypoxia for 72 h. Possibly, hypoxia may interfere with beneficial effects of SCFA on intestinal barrier function. As mentioned previously this study has several limitations including the type of enteroids used (duodenal vs. colonic) as well as time points collected. Additional studies are needed to elucidate mechanisms and determine whether SCFA may attenuate hypoxia-induced intestinal barrier dysfunction in *in vivo* models.

## Data Availability

The original contributions presented in the study are included in the article/Supplementary Material, further inquiries can be directed to the corresponding author.
