# Supplementary material for: Effects of short-chain fatty acids on intestinal function in an enteroid model of hypoxia
Source: Front Physiol. 2022 Dec 5;13:1056233. doi: 10.3389/fphys.2022.1056233 (PMC9760830; doi:10.3389/fphys.2022.1056233)
Supplement: Supplementary file 1 [file DataSheet1.docx]

| **Supplemental Table 1. Effects of butyrate (BUT) and acetate (ACET) on protein expression during 72-hr exposure to normoxia (CON) or hypoxia (HYP).** | | | | | | | | | | |
| --- | --- | --- | --- | --- | --- | --- | --- | --- | --- | --- |
|  | **Mean** | | | | | | | | | |
|  | **CON** | **HYP** | **CON+BUT** | **CON+ACET** | **HYP+BUT** | **HYP+ACET** | **HYP+BUT(24hr)** | **HYP+ACETT(24hr)** | **HYP+BUT(48hr)** | **HYP+ACET(48hr)** |
| Ghrelin | 1294.2 ± 205.6 | 1097.9 ± 174.1 | 1322.1 ± 229.5 | 1167.6 ± 291.0 | 1311.0 ± 225.2 | 1266.3 ± 194.2 | 985.2 ± 175.1 | 1276.8 ± 348.4 | 1069.7 ± 197.2 | 815.5 ± 175.5 |
| GLP_1 | 4.67 ± 0.84 | 5.04 ± 0.50 | 4.92 ± 1.52 | 3.49 ± 1.37 | 5.16 ± 1.35 | 3.16 ± 0.26 | 3.41 ± 0.45 | 2.19 ± 0.45 | 3.83 ± 0.38 | 3.41 ± 0.78 |
| PYY | 1.04 ± 0.17 | 0.75 ± 0.12 | 0.91 ± 0.09 | 0.79 ± 0.20 | 0.89 ± 0.09 | 0.83 ± 0.00 | 0.78 ± 0.11 | 0.89 ± 0.15 | 0.61 ± 0.11 | 0.47 ± 0.12 |
| IL_1b | 1.41 ± 0.38 | 0.87 ± 0.20 | 1.35 ± 0.17 | 1.34 ± 0.18 | 1.39 ± 0.21 | 0.98 ± 0.06 | 1.30 ± 0.16 | 1.26 ± 0.40 | 0.76 ± 0.18 | 0.72 ± 0.12 |
| IL_4 | 5.45 ± 0.42 | 4.66 ± 0.68 | 4.75 ± 0.57 | 5.04 ± 1.0 | 6.29 ± 0.79 | 5.45 ± 0.29 | 4.75 ± 0.58 | 4.33 ± 0.24 | 3.40 ± 0.31 | 2.92 ± 0.60 |
| IL_6 | 3.14 ± 0.38 | 2.63 ± 0.31 | 3.17 ± 0.68 | 2.75 ± 0.42 | 3.29 ± 0.25 | 2.46 ± 0.29 | 2.50 ± 0.48 | 2.62 ± 0.49 | 2.50 ± 0.31 | 1.86 ± 0.30 |
| IL_8 | 8667.1 ± 2191.9 | 1957.8 ± 601.5 | 12151.3 ± 823.7 | 10950 ± 774 | 10600 ± 465 | 4928.8 ± 499.5 | 11036.4 ± 663.0 | 10777.7 ± 1530.6 | 1369.6 ± 302.4 | 1005.7 ± 95.8 |
| IL_10 | 0.10 ± 0.01 | 0.05 ± 0.01 | 0.06 ± 0.00 | 0.05 ± 0.02 | 0.06 ± 0.01 | 0.05 ± 0.02 | 0.05 ± 0.01 | 0.05 ± 0.01 | 0.04 ±0.01 | 0.03 ± 0.01 |
| IL_12p70 | 0.58 ± 0.16 | 0.51 ± 0.12 | 0.62 ± 0.12 | 0.51 ± 0.20 | 0.67 ± 0.10 | 0.40 ± 0.13 | 0.60 ± 0.08 | 0.38 ± 0.05 | 0.34 ± 0.08 | 0.25 ± 0.07 |
| IL_17 | 0.24 ± 0.06 | 0.12 ± 0.02 | 0.27 ± 0.05 | 0.24 ± 0.07 | 0.27 ± 0.03 | 0.18 ± 0.03 | 0.26 ± 0.04 | 0.25 ± 0.06 | 0.10 ± 0.02 | 0.07 ± 0.02 |
| IL_18 | 3.22 ± 0.90 | 2.76 ± 0.48 | 2.19 ± 0.39 | 2.44 ± 0.17 | 4.62 ± 0.68 | 2.40 ± 0.42 | 3.81 ± 0.99 | 2.64 ± 0.36 | 1.56 ± 0.23 | 1.55 ± 0.33 |
| IFNγ | 2.66 ± 0.28 | 2.51 ± 0.17 | 2.66 ± 0.56 | 2.38 ± 0.32 | 2.66 ± 0.38 | 2.04 ± 0.00 | 2.38 ± 0.21 | 1.92 ± 0.64 | 1.94 ± 0.15 | 1.84 ± 0.32 |
| MCP_1 | 901.6 ± 303.5 | 194.8 ± 28.9 | 176.9 ± 89.8 | 85.2 ± 15.9 | 50.4 ± 8.7 | 126.2 ± 9.1 | 20.1 ± 1.7 | 96.2 ± 52.6 | 41.2 ± 20.1 | 48.7 ± 8.5 |
| Fas_L | 2.40 ± 0.41 | 2.29 ± 0.36 | 2.14 ± 0.29 | 2.09 ± 0.20 | 2.64 ± 0.48 | 2.48 ± 0.30 | 2.09 ± 0.16 | 2.38 ± 0.29 | 2.06 ± 0.14 | 1.74 ± 0.20 |
| Galectin_3 | 17536 ± 8762.8 | 29612 ± 6938.7 | 24519 ± 19265.3 | 11723 ± 699.1 | 109515 ± 7872.2 | 30442 ± 5022.3 | 81934 ± 11943.0 | 172472 ± 118659.3 | 13328 ± 2036.7 | 17840 ± 2294.0 |
| LDH_B | 1444.3 ± 1194.6 | 367.2 ± 47.5 | 660.2 ± 88.8 | 1087.4 ± 477.8 | 931.4 ± 75.3 | 2614.1 ± 1670.4 | 647.9 ± 48.3 | 1598.5 ± 896.0 | 407.6 ± 30.12 | 360.5 ± 33.04 |
| (24hr), cells pre-treated with BUT or ACET for 24hr before exposure; (48hr), cells pre-treated with BUT or ACET for 48hr before exposure. | | | | | | | | | | |

| **Supplemental Table 2. Effects of butyrate (BUT) and acetate (ACET) on gene expression during 72-hr exposure to normoxia (CON) or hypoxia (HYP).** | | | | | | | | | | |
| --- | --- | --- | --- | --- | --- | --- | --- | --- | --- | --- |
|  | **Mean** | | | | | | | | | |
|  | **CON** | **HYP** | **CON+BUT** | **CON+ACET** | **HYP+BUT** | **HYP+ACET** | **HYP+BUT(24hr)** | **HYP+ACETT(24hr)** | **HYP+BUT(48hr)** | **HYP+ACET(48hr)** |
| **RAB17** | 1.00 ± 0.115 | 0.717 ± 0.181 | 1.228 ± 0.575 | 1.523 ± 0.266 | 0.714 ± 0.077 | 0.451 ± 0.033 | 0.768 ± 0.116 | 0.627 ± 0.087 | 1.236 ± 0.223 | 0.891 ± 0.198 |
| **CLDN4** | 1.00 ± 0.064 | 0.757 ± 0.137 | 0.420 ± 0.289 | 0.409 ± 0.264 | 0.066 ± 0.066 | 0.047 ± 0.023 | 0.519 ± 0.076 | 0.099 ± 0.049 | 0.671 ± 0.155 | 0.479 ± 0.222 |
| **CHGA** | 1.00 ± 0.251 | 1.963 ± 1.044 | 1.362 ± 0.229 | 1.664 ± 0.512 | 2.010 ± 0.426 | 1.516 ± 0.556 | 1.815 ± 0.538 | 1.211 ± 0.404 | 4.184 ± 0.461 | 2.864 ± 0.507 |
| **DCLK1** | 1.00 ± 0.129 | 2.218 ± 1.163 | 1.401 ± 0.235 | 1.988 ± 0.569 | 2.300 ± 0.295 | 1.583 ± 0.537 | 1.837 ± 0.603 | 1.176 ± 0.328 | 4.256 ± 0.522 | 2.990 ± 0.448 |
| **IL_22** | 1.00 ± 0.227 | 2.152 ± 1.132 | 1.440 ± 0.331 | 1.910 ± 0.667 | 2.290 ± 0.464 | 1.543 ± 0.553 | 1.961 ± 0.572 | 1.158 ± 0.413 | 4.483 ± 0.978 | 3.112 ± 0.442 |
| **ALPI** | 1.000 ± 0.169 | 2.211 ± 1.003 | 1.612 ± 0.337 | 1.934 ± 0.710 | 2.611 ± 0.524 | 1.653 ± 0.553 | 1.891 ± 0.551 | 1.208 ± 0.338 | 4.568 ± 0.567 | 3.125 ± 0.598 |
| **PYY** | 1.00 ± 0.194 | 2.427 ± 1.335 | 1.546 ± 0.347 | 2.117 ± 0.598 | 2.572 ± 0.215 | 1.822 ± 0.676 | 2.092 ± 0.681 | 1.350 ± 0.481 | 5.041 ± 0.849 | 3.529 ± 0.581 |
| **IL_8** | 1.00 ± 0.031 | 0.762 ± 0.142 | 6.585 ± 5.643 | 4.406 ± 1.051 | 1.810 ± 0.884 | 2.811 ± 1.239 | 0.454 ± 0.116 | 3.697 ± 1.638 | 0.769 ± 0.155 | 0.457 ± 0.135 |
| **CLDN3** | 1.00 ± 0.069 | 1.328 ± 0.189 | 0.960 ± 0.376 | 1.068 ± 0.376 | 0.836 ± 0.172 | 0.736 ± 0.361 | 1.324 ± 0.187 | 0.673 ± 0.051 | 1.724 ± 0.445 | 1.218 ± 0.356 |
| **SLC16A1** | 1.00 ± 0.097 | 0.173 ± 0.017 | 0.387 ± 0.191 | 0.519 ± 0.286 | 0.256 ± 0.235 | 0.188 ± 0.090 | 0.170 ± 0.019 | 0.132 ± 0.033 | 0.178 ± 0.038 | 0.130 ± 0.033 |
| **SLC16A3** | 1.00 ± 0.018 | 0.198 ± 0.016 | 1.280 ± 0.524 | 1.399 ± 0.910 | 0.103 ±0.025 | 0.124 ± 0.030 | 0.256 ± 0.044 | 0.217 ± 0.127 | 0.341 ± 0.057 | 0.243 ± 0.063 |
| **TFF3** | 1.00 ± 0.048 | 0.592 ± 0.047 | 0.114 ± 0.029 | 0.136 ± 0.019 | 0.331 ± 0.034 | 0.358 ± 0.221 | 0.410 ± 0.051 | 0.089 ± 0.008 | 0.669 ± 0.138 | 0.474 ± 0.199 |
| **TGFB1** | 1.00 ± 0.204 | 1.264 ± 0.112 | 0.332 ± 0.136 | 0.570 ± 0.148 | 0.404 ± 0.075 | 0.357 ± 0.196 | 1.268 ± 0.087 | 0.558 ± 0.102 | 1.433 ± 0.186 | 0.813 ± 0.310 |
| **CDH1** | 1.00 ± 0.051 | 0.478 ± 0.040 | 0.791 ± 0.358 | 0.946 ± 0.407 | 0.585 ± 0.052 | 0.862 ± 0.484 | 0.277 ± 0.019 | 0.439 ± 0.040 | 0.312 ± 0.096 | 0.204 ± 0.092 |
| **LYZ** | 1.00 ± 0.088 | 0.748 ± 0.050 | 0.486 ± 0.163 | 0.812 ± 0.377 | 0.946 ± 0.145 | 0.761 ± 0.406 | 0.607 ± 0.067 | 0.217 ± 0.021 | 0.453 ± 0.091 | 0.360 ± 0.178 |
| **FAS** | 1.00 ± 0.149 | 0.512 ± 0.138 | 0.283 ± 0.045 | 0.400 ± 0.136 | 0.407 ± 0.064 | 0.317 ± 0.142 | 0.381 ± 0.090 | 0.199 ± 0.066 | 0.745 ± 0.125 | 0.565 ± 0.092 |
| **HSPA1A** | 1.00 ± 0.051 | 0.094 ± 0.016 | 1.524 ± 0.630 | 1.273 ± 0.850 | 0.123 ± 0.050 | 0.147 ± 0.147 | 0.134 ± 0.016 | 0.096 ± 0.029 | 0.066 ± 0.013 | 0.049 ± 0.018 |
| **HSF1** | 1.00 ± 0.070 | 1.388 ± 0.395 | 1.160 ± 0.272 | 1.384 ± 0.141 | 1.580 ± 0.408 | 1.693 ± 0.791 | 1.288 ± 0.102 | 1.121 ± 0.176 | 2.208 ± 0.189 | 1.606 ± 0.353 |
| **ACADM** | 1.00 ± 0.030 | 0.453 ± 0.049 | 0.708 ± 0.312 | 0.632 ± 0.235 | 0.555 ± 0.087 | 0.474 ± 0.184 | 0.522 ± 0.049 | 0.234 ± 0.035 | 0.673 ± 0.135 | 0.477 ± 0.098 |
| **OCLN** | 1.00 ± 0.135 | 1.052 ± 0.045 | 0.487 ± 0.146 | 0.555 ± 0.225 | 0.388 ± 0.097 | 0.372 ± 0.124 | 0.679 ± 0.087 | 0.397 ± 0.151 | 1.069 ± 0.259 | 0.869 ± 0.360 |
| **SLC5A8** | 1.00 ± 0.233 | 2.402 ± 1.231 | 1.592 ± 0.289 | 2.021 ± 667 | 2.522 ± 0.508 | 1.725 ± 0.516 | 2.138 ± 0.695 | 1.283 ± 0.457 | 5.110 ± 0.861 | 3.526 ± 0.690 |
| **TJP1** | 1.00 ± 0.100 | 0.604 ± 0.168 | 1.445 ± 0.660 | 1.123 ± 0.365 | 0.862 ± 0.212 | 0.778 ± 0.442 | 0.507 ± 0.120 | 0.639 ± 0.179 | 0.908 ± 0.153 | 0.641 ± 0.146 |
| **FFAR2** | 1.00 ± 0.236 | 2.293 ± 1.332 | 1.656 ± 0.345 | 2.152 ± 0.673 | 2.509 ± 0.296 | 1.853 ± 0.490 | 2.139 ± 0.714 | 1.442 ± 0.440 | 5.428 ± 1.037 | 3.585 ± 0.693 |
| **MYO7B** | 1.00 ± 0.143 | 1.082 ± 0.544 | 0.716 ± 0.134 | 0.877 ± 0.287 | 1.074 ± 0.163 | 0.835 ± 0.237 | 0.966 ± 0.314 | 0.537 ± 0.167 | 2.188 ± 0.249 | 1.479 ± 0.331 |
| **FABP2** | 1.00 ± 0.203 | 0.308 ± 0.171 | 0.223 ± 0.171 | 0.268 ± 0.083 | 0.343 ± 0.070 | 0.249 ± 0.082 | 0.286 ± 0.093 | 0.172 ± 0.061 | 0.669 ± 0.095 | 0.452 ± 0.075 |
| (24hr), cells pre-treated with BUT or ACET for 24hr before exposure; (48hr), cells pre-treated with BUT or ACET for 48hr before exposure. | | | | | | | | | | |
